# New Appliances and Things Medical

**Published:** 1906-02-17

**Authors:** 


### NEW APPLIANCES AND THINGS MEDICAL.

### COLEMAN'S WINCARNIS.

(Messrs. Coleman and Co., Ltd., Wincarnis Works, Norwich.)

We have received a sample of this well-known preparation—which we have tried. As is probably well known, Coleman's Wincarnis consists of wine, Liebig's extract of meat, and malt extract. It will thus be seen that it possesses stimulant as well as nutritive properties; that, in short, it may be considered a food as well as a medicine. The potential energy which it as a food contains is of a very labile form, and can be converted into kinetic biological energy in case of greatly impaired digestive power. Although it may be used with advantage by the healthy, we think it pre-eminently suited as a nutrient and stimulant for the sick.

### KUDOS COCOA ESSENCE AND MILK CHOCOLATE.

(The Bovril Company, Limited, Old St., London, E.C.)

We have received a specimen of Kudos chocolate and cocoa essence. Chocolate consists essentially of ground cocoa from which only a small quantity of the natural cocoa fat has been removed, and to which some sugar and often some flavouring agents have been added. Chocolate should melt easily in the mouth and should be of a clear brown colour, and not present what is called a "bloom." The chocolate before us presents these characteristics and is a good preparation and to be recommended. Kudos cocoa is a concentrated preparation of cocoa of which half a teaspoonful is required to make a breakfast-cupful of the beverage. It has a good aroma and has been largely freed from fat, and contains an average amount of the alkaloid.

